# Affective touch enhances low gamma activity during hand proprioceptive perception in children with different neurodevelopmental conditions

**DOI:** 10.3389/fnhum.2025.1538428

**Published:** 2025-02-11

**Authors:** Álvaro Sabater-Gárriz, José Antonio Mingorance, Francesc Mestre-Sansó, Vicent Canals, Yannick Bleyenheuft, Pedro Montoya, Inmaculada Riquelme

**Affiliations:** ^1^Research Institute on Health Sciences (IUNICS), University of the Balearic Islands, Palma de Mallorca, Spain; ^2^Department of Nursing and Physiotherapy, University of the Balearic Islands, Palma de Mallorca, Spain; ^3^Health Research Institute of the Balearic Islands (IdISBa), Palma, Spain; ^4^Balearic ASPACE Foundation, Marratxí, Spain; ^5^Industrial Engineering and Construction Department, University of the Balearic Islands, Palma, Spain; ^6^Institute of Neuroscience, Université catholique de Louvain, Brussels, Belgium

**Keywords:** autism, cerebral palsy, gamma oscillations, affective touch, proprioception, sensorimotor integration

## Abstract

**Background:**

Gamma wave activity in the sensorimotor cortex is a critical neural mechanism associated with proprioceptive processing, which is essential for motor coordination, balance, and spatial orientation. The modulation of gamma oscillations by different types of tactile stimuli, including affective touch, is not well understood, particularly in children with neurodevelopmental disorders such as cerebral palsy and autism spectrum disorder.

**Aims:**

This study aims to explore how affective touch influences gamma oscillatory activity and proprioceptive performance in children with typical development, cerebral palsy and autism spectrum disorders.

**Methods and procedures:**

EEG data were recorded from participants during passive wrist mobilizations under three conditions: following an affective touch stimulus, after a non-affective touch stimulus, and with no tactile stimulation. Time-frequency analysis of low gamma activity (30–45 Hz) on the left somatosensory cortex was conducted for each condition. Proprioceptive performance was assessed through participants’ accuracy in identifying wrist positions. Proprioception and pleasantness of affective and non-affective touch were also assessed.

**Results:**

Affective touch increased proprioceptive gamma power density. Children with cerebral palsy had poorer proprioception and higher brain gamma power density for processing movement than children with typical development or autism, and their proprioception worsened with non-affective touch.

**Conclusion and implications:**

These findings highlight the potential of affective touch to modulate gamma oscillatory activity and enhance proprioceptive function, particularly in children with cerebral palsy. The results underscore the importance of incorporating emotionally meaningful sensory inputs in therapeutic interventions to support proprioceptive and motor function in children with neurodevelopmental disorders.

## 1 Introduction

Proprioception, the body’s ability to sense limb position and the effort required to maintain it, is fundamental to motor coordination, balance, and spatial awareness, enabling individuals to interact with their environment fluidly and effectively (Proske and Gandevia, 2012). Deficits in proprioceptive function can severely impact daily activities, limiting independence and quality of life, particularly in individuals with motor and neurodevelopmental disorders (Volkan-Yazici et al., 2018).

Gamma wave activation in the brain is increasingly recognized as a key neural mechanism underpinning proprioceptive processing, particularly during tasks that involve sensing body position and movement (Arnfred et al., 2007; Tatti et al., 2022; Albanese et al., 2023). Evidence suggests that proprioceptive stimuli, such as weight changes in a handheld load, can evoke gamma oscillations in the parietal cortex contralateral to the stimulated side, indicating an early somatosensory feature integration process (Arnfred et al., 2007). Additionally, gamma oscillations in the primary motor cortex have been observed during self-paced motor tasks, reinforcing their role in voluntary motor control and sensory feedback (Cheyne et al., 2008; Muthukumaraswamy, 2010). The activation of fast-spiking interneurons at gamma frequencies appears essential for sensory processing and attention, supporting the importance of gamma waves in proprioceptive and sensory gating functions (Gaetz et al., 2013; Spooner et al., 2020). Collectively, these findings highlight the significance of gamma oscillations in integrating sensory and motor functions during proprioceptive tasks.

In neurodevelopmental disorders such as cerebral palsy (CP) and autism spectrum disorder (ASD), gamma oscillations are often altered, reflecting challenges in sensory-motor integration and proprioception. Studies indicate that individuals with CP tend to show reduced or dysregulated gamma activity during motor and proprioceptive tasks, suggesting a dysfunction in neuronal synchronization that impacts movement planning and execution (Busboom et al., 2024; Hoffman et al., 2019). This reduction in gamma activity may contribute to the proprioceptive difficulties characteristic of CP, as gamma oscillations are involved in encoding sensory information and coordinating motor responses (Muthukumaraswamy, 2010; Shin and Moore, 2019). Similarly, in individuals with ASD, disruptions in gamma oscillatory activity have been linked to deficits in sensory processing and attention, underscoring the importance of gamma waves in sensory integration and focus modulation (Kakuszi et al., 2023; Jia et al., 2021). These findings have driven research into therapeutic interventions targeting sensory modulation, particularly focusing on enhancing gamma activity as a means of improving proprioception and motor control (Perfetti et al., 2011).

Affective touch, characterized by gentle, caress-like tactile stimulation, has been shown to enhance proprioceptive accuracy and body awareness in neurotypical individuals (Crucianelli et al., 2013). This form of touch engages C-tactile (CT) afferents, which are linked to the insular cortex and contribute to the emotional and social aspects of tactile perception (McGlone et al., 2014). Research has shown that affective touch enhances sensory integration and contributes to body awareness and emotional regulation (Sacchetti et al., 2021). Although there is limited direct evidence linking affective touch with gamma oscillatory activity, gamma waves are well-documented in their role in sensory-motor integration, attentional processes, and the facilitation of cognitive functions (Tallon-Baudry, 2009). This suggests that the pathways activated by affective touch could potentially engage mechanisms that support gamma activity, thereby influencing sensory processing and attention indirectly. Given that both CP and ASD present with associated proprioceptive deficits and atypical gamma activity, investigating whether affective touch could influence gamma oscillations and improve proprioceptive integration in these populations could provide valuable insights.

In this study, we examined proprioceptive processing, specifically focusing on gamma wave activity, by recording EEG responses during passive wrist mobilizations in three groups: typically developing children (TD), children with ASD, and children with CP. Participants experienced three proprioceptive conditions: without tactile stimulation, after an affective touch stimulus and after a non-affective touch stimulus. This approach allowed us to assess how proprioceptive processing, as reflected in gamma oscillatory activity, differs across groups and conditions, highlighting the influence of affective touch on proprioceptive integration. Given the established relevance of the low gamma band (30–45 Hz) in sensory and motor integration, particularly for proprioceptive tasks (Patino et al., 2008; Crone et al., 1998; Aoki et al., 2023), this study focused specifically on this frequency range. The low gamma range has been linked to sensory-motor coordination, essential for proprioceptive processing. Additionally, the low gamma frequencies are less susceptible to high-frequency noise and artifacts, thus offering more stable and reliable EEG recordings (Nottage and Horder, 2016). We hypothesized that affective touch might increase low gamma activation in individuals with neurodevelopmental conditions (CP and ASD), enhancing proprioceptive brain processing as it occurs in neurotypical population (Crucianelli et al., 2013).

## 2 Materials and methods

### 2.1 Participants

Typically developing children and adolescents (TD), ranging from 6 to 19 years old, were recruited from various educational institutions in Majorca, Spain. A total of 21 children agreed to participate, with parental consent obtained for each (mean age = 10.66 years, SD = 4.73; 10 girls). Similarly, children with neurodevelopmental disorders within the same age range were recruited through patient associations and educational centers in Majorca. Twenty children with ASD (mean age = 13.25 years, SD = 3.86; 4 girls) and 14 children with CP (mean age = 16.00 years, SD = 4.15; 7 girls) participated in the study. Table 1 provides an overview of the clinical characteristics of participants with neurodevelopmental disorders. Parents signed informed consent and children gave oral consent to participate. The study received ethical approval from the Ethics Committee of the ASPACE Balearic Foundation and the Research Ethics Committee at the University of the Balearic Islands (ref. 127CER19).

**Table 1:** Clinical characteristics of children with neurodevelopmental disorders (children with autism spectrum disorders and children with cerebral palsy).

Variable (n)	Children with autism spectrum disorders (*n* = 20)	Children with cerebral palsy (*n* = 14)
Cognitive function		
Normal cognition	19	10
Mild impairment	1	4
Moderate impairment	0	0
Language function		
Fluid language	20	12
Some sentences or echolalia	0	2
Some words	0	0
Non verbal	0	0
Gross motor function classification system		
Level I	20	1
Level II	0	4
Level III	0	5
Level IV	0	2
Level V	0	2
Manual ability function classification system		
Level I	20	4
Level II	0	2
Level III	0	3
Level IV	0	3
Level V	0	2

### 2.2 Nottingham sensory assessment

The Nottingham Sensory Assessment is a standardized tool commonly used to assess sensory deficits, including proprioception, in individuals with neurological impairments (Lincoln et al., 1998). The Nottingham Sensory Assessment evaluates various sensory modalities, such as light touch, pressure, temperature, and proprioception, with proprioception specifically tested by passively moving the joints and asking the participant to identify the direction or final position of the movement. In the present study, the wrist was randomly and sequentially positioned in palmar flexion, dorsal flexion, radial deviation or ulnar deviation. Proprioception was scored according to the following criteria: 2 = normal, ability to describe final joint position within 10° range of error; 1 = partially impaired, ability to appreciate joint movement but failure to detect movement direction; 0 = impaired, no appreciation of joint movement. The final score was the sum of the four scores of the different positions. A higher score on the test indicated better proprioception (range 0–16). This procedure has been used previously in children with ASD and children with CP (Riquelme et al., 2024).

### 2.3 EEG recording and data processing

Electroencephalography (EEG) data were collected from 32 scalp electrodes (ECI; Nieuwkoop, The Netherlands) positioned according to the international 10/20 system. Reference electrodes were placed on both mastoids, and impedance levels were maintained below 10 kΩ. Recordings were made using a BrainAmp amplifier (Brain Products, Inc., Munich, Germany) with a sampling rate of 1,000 Hz, applying a high-pass filter at 0.10 Hz, a low-pass filter at 70 Hz, and a notch filter at 50 Hz.

EEG data were continuously recorded during wrist passive flexion or extension movements. A specially designed mechanical arthromotor device passively and repeatedly moved the right wrist of the participant, in a random order, at a speed of 9°/s, and the remained in the final position (movement angle of 36°). The arm of the children was covered with a pannel for preventing the children’s view. After the hand movement, a picture of two hands, one with flexion and one with extension of the wrist, was displayed on the screen and the children were instructed to report their hand position by pressing a button within a 5-s window. Marks in the EEG recording allowed to offline examine the proprioception hits by comparing the position of the arthomotor and the button pressed by the children in each image. Movements were performed in three tactile conditions, presented in blocks: no touch, affective touch and non-affective touch.

For tactile stimulation, children received manual brush strokes to the dorsal surface of their right forearm using a soft cosmetic brush (3 cm of diameter). Two lines, 9 cm apart, were demarcated on the dorsal surface of their right forearm. An auditory metronome (via headphones) guided the examiner in delivering the brush strokes in a proximal-distal direction at each of two velocities: 3 cm/s for affective touch, and 30 cm/s for non-affective touch. For stimuli delivered at 3 cm/s the distance was covered once per 3 s (9 cm stroking area × 3 cm/s), while for stimuli delivered at 30 cm/s distance was covered 3 times per second (10 cm stroking area × 30 cm/s). In the non-touch condition, no tactile stimulus was provided and children only experienced the proprioceptive stimuli provided by the arthromotor. Affective and non-affective stimuli were repeated off-line with the same parameters and children scored the pleasantness (1 very unpleasant – 10 very pleasant) and intensity (1 very weak – 10 very strong) of the stimulus in 5 trials per condition; the mean of the 5 scores was taken as the score of pleasantness and intensity in each condition.

**Figure 1:**
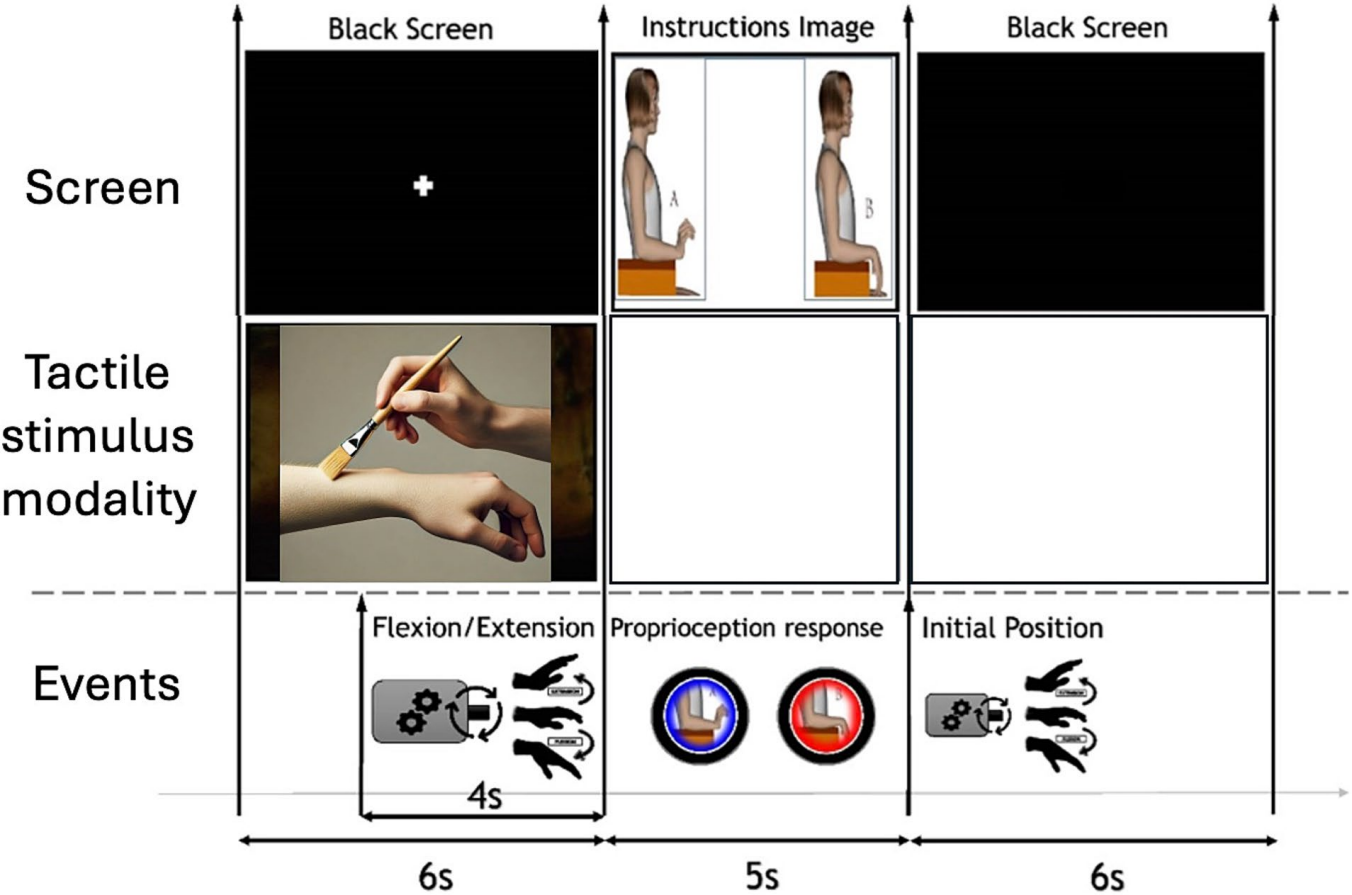
Time sequence of the experimental task.

The EEG task started by providing tactile stimulation (or no touch) while the computer screen showed a fixation cross; tactile stimulation persisted for 6 s, until the picture of the choosing hands appeared. After 2 s from the beginning of the tactile stimulus, the arthromotor started the passive movement of the hand; that implies that tactile stimulation started before the movement and reminded until the artromothor finished the passive movement. After this, a 5-s section started, where the children had to choose the hand position. Finally, the arthromotor returned the hand to its initial position and a 6-s interstimulus interval allowed the children relaxing until the following trial. The time sequence of the task is displayed in Figure 1. Each block was composed by 15 trials following the same sequence. The order of the blocks was randomized across participants to mitigate order effects. Further details on the methodology of the task can be found in Mestre-Sansó et al. (2024).

The EEG recordings of 2 children with TD, 1 child with ASD and 2 children with CP were discarded due to excessive artifacts that prevented further statistical analysis. EEG was segmented offline in epochs of 2000 ms duration (from −2000 ms to 0 ms relative to tactile stimuli and from 1,000 ms to 3,000 ms relative to wrist movement onset). EEG epochs were baseline corrected and digitally filtered (0.05–45 Hz band pass filter) and corrected for eye-movement artifacts by using Gratton regression method. Furthermore, EEG epochs containing artifacts (maximal allowed voltage step/sampling point = 100 μV, minimal allowed amplitude = −100 μV, maximal allowed amplitude = 100 μV, or maximal allowed absolute difference in the epoch = 100 μV) were automatically rejected. In addition, an Independent Component Analysis (ICA) was performed to explore potential remaining eye movement artifacts, where frontal components were visually inspected and manually removed. Only recordings of left hemispheric electrodes were used in further analyses, as movement was applied on the right wrist.

### 2.4 Time–frequency analysis of EEG data

Time-frequency analysis was performed for each subject using the FieldTrip software toolbox version 20,230,503 (Donders Institute for Brain, Cognition and Behaviour, Radboud University Nijmegen) for MATLAB, taking an average of the 15-trial 2-s segments from baseline (2 s previous to the tactile stimuli) and from the 2-s segments between +1 to +3 s relative to the onset of passive wrist movements on each condition: no touch, affective touch, and non-affective touch. An analysis of the left somatosensory cortex, considering C3, CP1, CP5, P7, P3 electrodes, was performed. The power density in the somatosensory area was obtained for the low gamma frequency band (30–45 Hz). The time-frequency analysis was conducted using MATLAB’s function “wltconvol,” applying baseline correction over the interval of [−0.2, 0.0] seconds, where cfg.baselinetype = ‘relative,’ expressing differences in relative terms. This approach was aimed to provide a robust and equitable comparison across subjects and conditions, to mitigate influences from external factors and ensuring that the differences observed and reported in our study are attributable to the tactile stimulus effects and experimental conditions, and not to artifactual signal variations or inherent differences among participants. Following this initial procedure, the low Gamma frequency band of 30–45 Hz was analyzed for each participant in each tactile stimulation condition. This analysis provided us with a power density value for each subject at intervals of 0.01 s.

### 2.5 Statistical analyses

The statistical package SPSS (V.22, IBM, Armonk, NY, United States) was used for statistical analyses. Descriptive statistics were used to characterize the sample and describe tactile stimulus ratings and proprioception hits. For assessing the tactile-related modulation of the low Gamma power spectrum, we utilized a General Linear Model (GLM). This approach allowed us to perform repeated measures analyses of variance (ANOVA) with GROUP (TD children vs. children with ASD vs. children with CP) as between-subject factor, and TIME (baseline vs. post-movement) and CONDITION (no touch vs. affective touch vs. non-affective touch) as within-subject factors. Additional ANOVAs with GROUP and CONDITION factors were used for examining differences in emotion pleasantness and intensity, as well as in proprioception hits. The factor TIME was not included in the ANOVAs examining emotional pleasantness, intensity, and proprioception because these variables were assessed at a single time point. Specifically, pleasantness and intensity ratings were collected immediately following the respective tactile stimuli, and proprioceptive performance was measured under stable conditions without temporal variation. Greenhouse–Geisser correction was applied for the violation of sphericity assumptions; Bonferroni corrections were applied for post-hoc comparisons.

## 3 Results

### 3.1 Wrist proprioception

**Figure 2:**
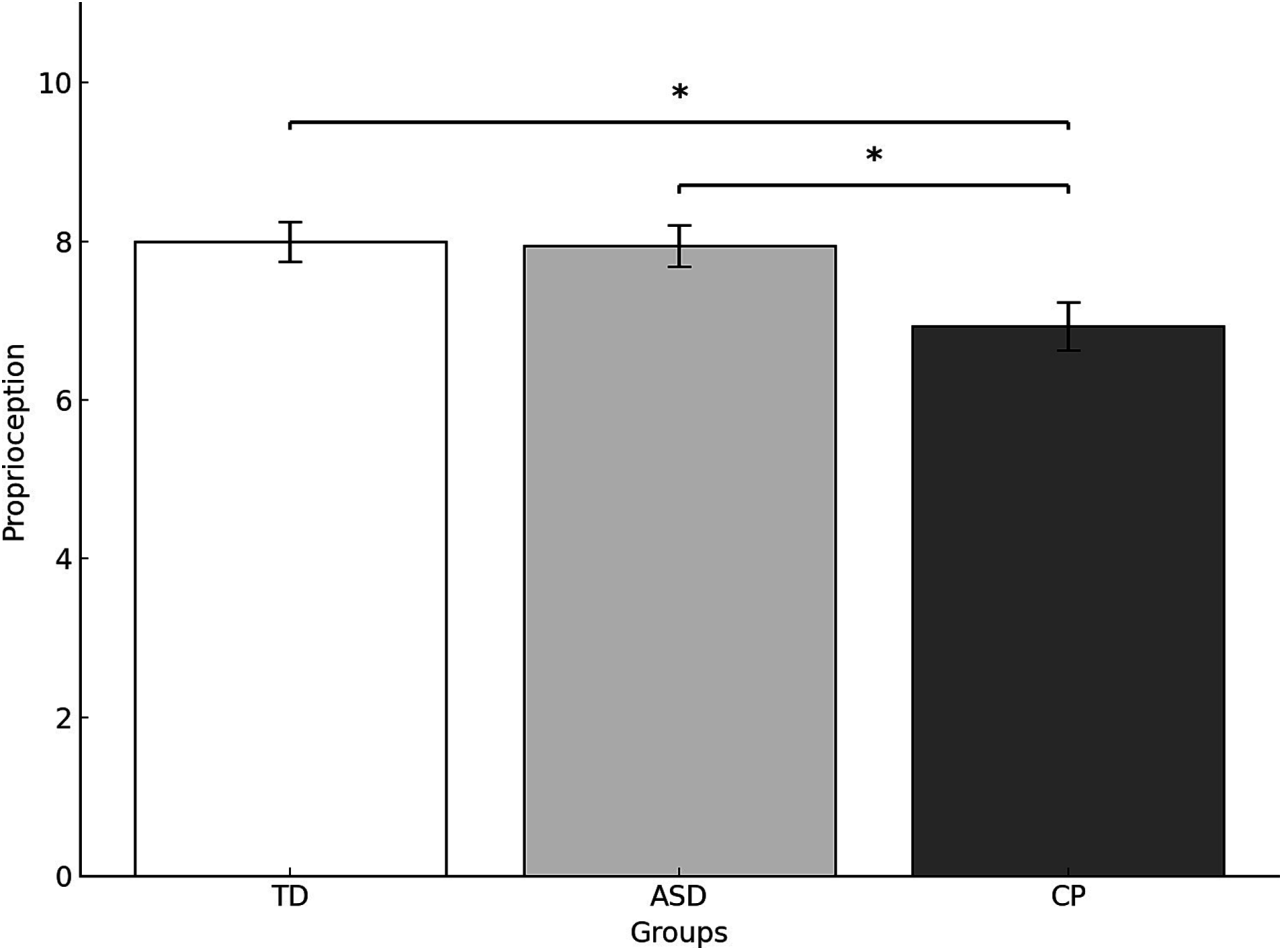
Proprioception scores (mean and standard deviation) by group. TD, Typically Developing children; ASD, children with Autism Spectrum Disorder; CP, children with Cerebral Palsy; *p < 0.05. Error bars represent the standard error of the mean (SEM).

ANOVA results revealed a significant difference in proprioception across groups (*F*(2,51) = 4.406, *p* = 0.017, $\eta_{p}^{2}=0.147$ ). *Post hoc* comparisons indicated that TD children and children with ASD scored significantly higher in proprioception than children with CP (*p* = 0.026 and *p* = 0.042, respectively). No significant difference was found between TD children and children with ASD (*F*(1,51) = 0.053,*p* = 0.818,ηp2 = 0.001F(1,51) = 0.053,p = 0.818, $\eta_{p}^{2}=0.001$ ) (Figure 2).

### 3.2 Pleasantness and intensity of affective and non-affective touch on the right forearm

**Figure 3:**
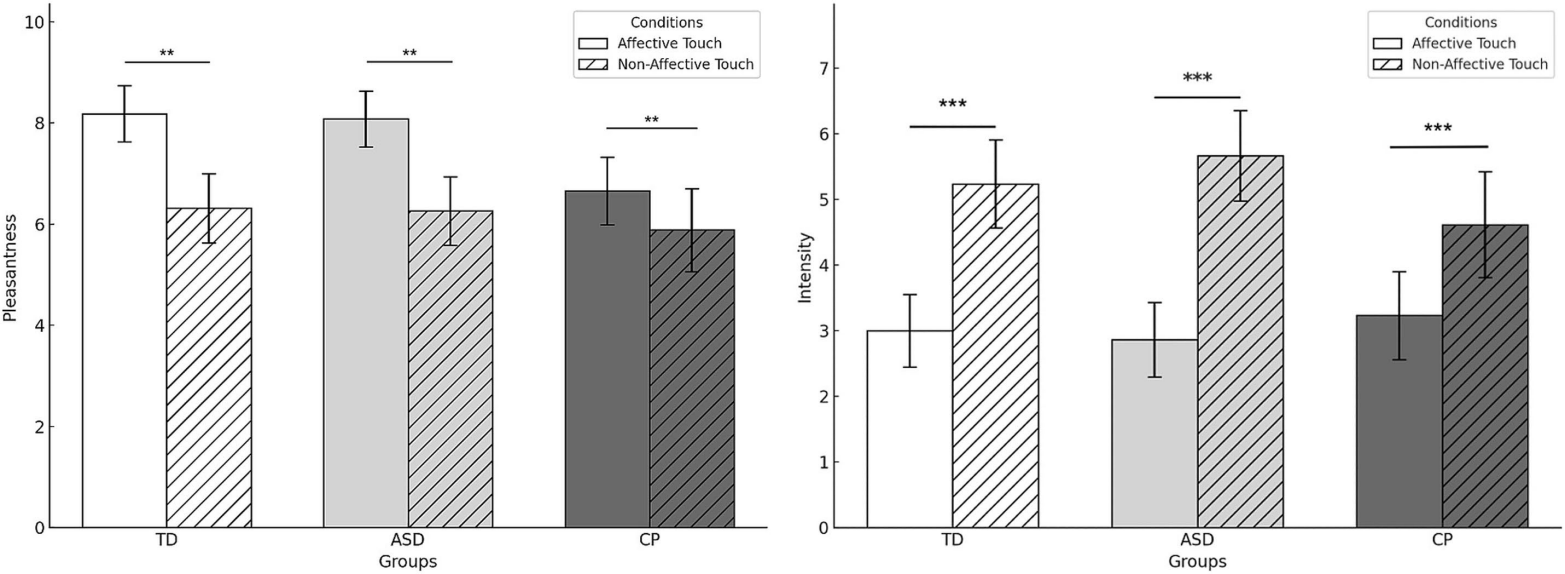
Pleasantness and intensity of affective and non-affective touch on the right forearm by group. TD, Typically Developing children; ASD, children with Autism Spectrum Disorder; CP, children with Cerebral Palsy; *p < 0.05. Error bars represent the standard error of the mean (SEM). Although the ANOVA revealed no significant CONDITION × GROUP interaction, data are presented by group to illustrate the main effect of CONDITION, which was consistent across all groups. **p < 0.01, ***p < 0.001.

Figure 3 displays the descriptive data of pleasantness and intensity for affective and non-affective touch in the three groups. In affective touch, ANOVA results revealed significant main effects of CONDITON both in the pleasantness and intensity scores (*F*(1,48) = 11.298, *p* = 0.002, $\eta_{p}^{2}=0.122$ and *F*(1,47) = 18.026, *p* < 0.001, $\eta_{p}^{2}=0.177$ respectively), with higher pleasantness and lower intensity for affective touch compared to non-affective touch. No significant main effect of GROUP was observed for pleasantness (*F*(2,48) = 1.584,*p* = 0.216, $\eta_{p}^{2}=0.062$ ) or intensity (*F*(2,47) = 1.257, *p* = 0.294, $\eta_{p}^{2}=0.051$ ). Similarly, no significant interaction effect of GROUP * CONDITION was found for pleasantness (*F*(2,48) = 1.113, *p* = 0.337, $\eta_{p}^{2}=0.044$ ) or intensity (*F*(2,47) = 0.837, *p* = 0.439, $\eta_{p}^{2}=0.034$ ).

### 3.3 Modulation of brain low gamma oscillations

A significant main effect of CONDITION was observed (*F*(2,46) = 4.841, *p* = 0.020, $\eta_{p}^{2}=0.093$ ), suggesting that the type of tactile stimulus influences gamma activation. Pairwise comparisons using Bonferroni correction showed that gamma activation was significantly higher in the affective touch condition compared to the non-affective touch condition (*p* = 0.028). There was no significant difference in gamma activation between affective touch and no touch (*p* = 0.301) or between no touch condition and non-affective touch (*p* = 0.193). No significant main effects of TIME (*F*(1,47) = 1.433, *p* = 0.237, $\eta_{p}^{2}=0.030$ ) or GROUP (*F*(2,47) = 0.269, *p* = 0.765, $\eta_{p}^{2}=0.011$ ) were found.

**Figure 4:**
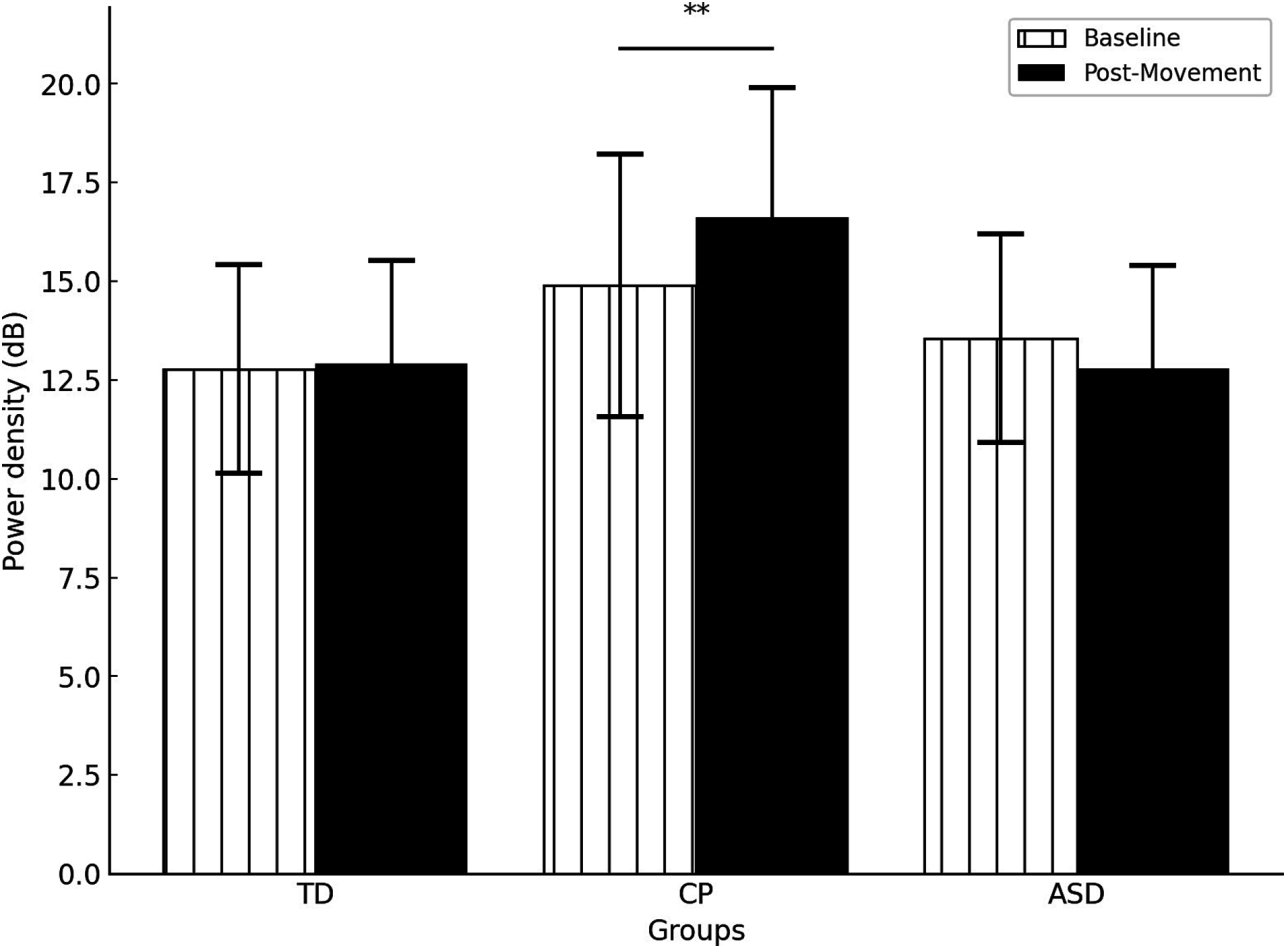
Gamma power density by group in the different times (Baseline vs Post movement). TD, Typically Developing children; ASD, children with Autism Spectrum Disorder; CP, children with Cerebral Palsy. **p = 0.007. Error bars represent the standard error of the mean (SEM).

A significant interaction GROUP * TIME (*F*(2,47) = 5.057, *p* = 0.010, $\eta_{p}^{2}=0.177$ ) indicated that the change in gamma activation from baseline to post-movement varies depending on the group. Specifically, *post hoc* tests indicated that only children with CP exhibited higher gamma power in post-movement than at baseline (*p* = 0.007), whereas no differences were observed in TD children or children with ASD (both *p* > 0.122) (Figure 4). No significant differences were observed between groups at either baseline or post-movement (all *p* > 0.05).

**Figure 5:**
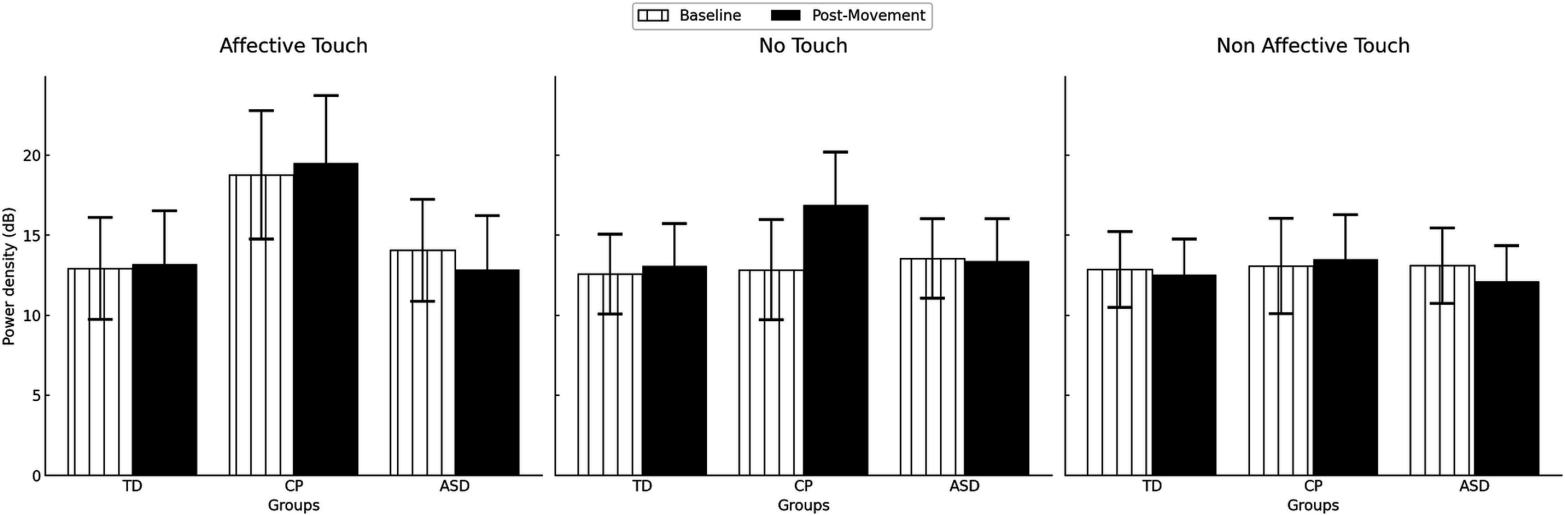
Gamma power density by group in the different times (Baseline vs Post movement) and conditions (No touch vs Affective touch vs Non-affective touch). TD, Typically Developing children; ASD, children with Autism Spectrum Disorder; CP, children with Cerebral Palsy. Error bars represent the standard error of the mean (SEM).

The interaction GROUP * CONDITION did not reach conventional levels of statistical significance (*F*(4,94) = 2.556, *p* = 0.067; $\eta_{p}^{2}=0.098$ ). However, this potential trend was explored to further explain how tactile stimulus effects on gamma activation could differ across groups. *Post hoc* analysis showed that, only within the CP group, proprioceptive gamma activation was significantly higher after affective touch compared to non-affective touch (*p* = 0.004), whereas no differences were found between other conditions (*p* = 0.258) or in TD children or children with ASD (all *p* > 0.714). There were no significant differences in gamma activation between groups within each tactile condition (all *p* > 0.844). Figure 5 displays the descriptive data of low gamma power density in each group, time and condition.

### 3.4 Proprioceptive hits

**Figure 6:**
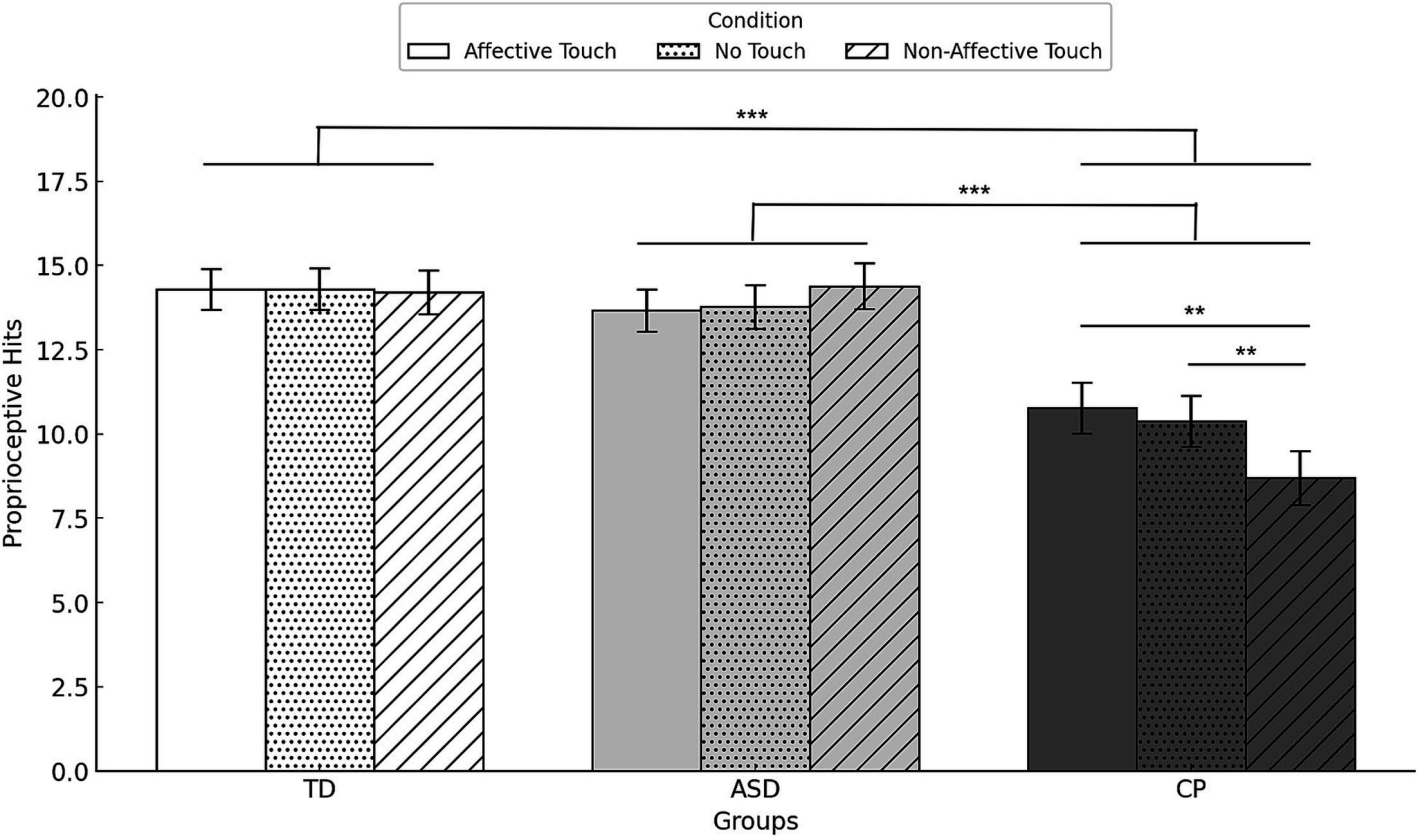
Mean proprioceptive hits with standard error by group and condition. TD, Typically Developing children; ASD, children with Autism Spectrum Disorder; CP, children with Cerebral Palsy. **p < 0.01, ***p < 0.001. Error bars represent the standard error of the mean (SEM).

Figure 6 displays the descriptive data of proprioceptive hits in each group and condition.

ANOVA results revealed a significant main effect of GROUP on proprioceptive hits (*F*(2,48) = 13.109, *p* < 0.001, $\eta_{p}^{2}=0.430$ ), indicating that children with CP had less proprioceptive hits than TD children (*p* < 0.001) or children with ASD (*p* < 0.001). No significant differences were found between TD children and children with ASD. No main effect of CONDITION was observed (*F*(2,48) = 1.760, *p* = 0.178, $\eta_{p}^{2}=0.035$ ). A significant GROUP * CONDITION (*F*(4,96) = 4.632, *p* = 0.002, $\eta_{p}^{2}=0.098$ ) revealed that children with CP had less proprioceptive hits that TD children and children with ASD in all touch conditions (all *p* < 0.015), whereas no differences were found between TD children and children with ASD. Within the CP group, proprioceptive hits in affective touch and no touch were higher than in non-affective touch (both *p* = 0.002). No significant differences were observed between the different conditions in TD children and children with ASD.

## 4 Discussion

The primary aim of this study was to investigate the effects of affective and non-affective tactile stimuli on proprioceptive performance and proprioceptive-elicited brain gamma activation in typically developing children (TD), autism spectrum disorder (ASD), and cerebral palsy (CP). In all the children, proprioceptive gamma power density was higher when it was simultaneous to affective touch than when passive movement perception was simultaneous to non-affective touch or not accompanied by any tactile stimulus. Children with CP had poorer proprioception than TD children or ASD and were the only group to show increment of gamma power density after passive movement. Children with CP also had more errors when identifying their wrist position than the other groups, and they had more errors when passive movement was associated to non-affective touch.

The findings of this study highlight the unique influence of affective touch on brain gamma activation elicited by passive movement and proprioceptive perception. The heightened gamma response when proprioceptive perception was simultaneous to affective touch in all children, regardless of their neurodevelopmental condition, may suggest that affective touch facilitates the cognitive resources needed to optimize proprioceptive performance (Miall et al., 2021), providing a form of sensory input that enhances neural engagement and integration. Previous findings from our lab have shown that emotional stimuli could influence brain’s processing of proprioception. On the other hand, brain seems especially sensitive to emotionally meaningful tactile stimuli, that may facilitate emotional and sensory integration (Gentsch et al., 2016; Olausson et al., 2016). Affective touch can engage C-tactile (CT) afferents, which are known for their role in enhancing sensory processing and integration related to body awareness (Crucianelli et al., 2013). While direct evidence linking CT afferents to proprioceptive modulation is limited, their influence on body ownership and sensory integration suggests that they could indirectly support proprioceptive processes. This effect seems to affect particularly to children with CP, that also showed that affective touch did not interfere their hand position hits, contrary to non-affective touch. In these children with poor proprioception, affective touch may have served as a mechanism aiding in recruiting the neural resources needed for effective proprioceptive perception (Howard et al., 2003; Wren et al., 2023), for example, helping sustain attention and working memory during proprioceptive processing (Busboom et al., 2024; Howard et al., 2003). These insights align with literature emphasizing that affective touch extends beyond a simple sensory experience; it acts as a multisensory input capable of influencing cognitive and motor processes by engaging neural networks involved in attention and sensory integration (Löken et al., 2009; Morrison, 2021). Such adaptations could be particularly relevant in children with CP, where altered sensory pathways might necessitate increased resources to achieve optimal functional performance (Wilson et al., 2016). Affective touch may have potential as an additional therapeutic agent to improve motor and sensory function in this population.

Interestingly, changes in gamma power density after movement were only observed in children with CP, suggesting that proprioceptive movement alone did not substantially modulate gamma activity in TD children and children with ASD. This finding might appear contradictory to previous evidence that indicates that proprioceptive stimuli can modulate gamma oscillations (Kanayama et al., 2009; Engel and Fries, 2010). One plausible explanation is that the modulation of gamma activity in response to proprioceptive input may be contingent upon the complexity and active engagement required by the task. While direct evidence linking this to proprioceptive tasks remains limited, previous research has shown that gamma oscillations are more prominently modulated during tasks that require higher cognitive engagement or involve complex motor activity (Howard et al., 2003; Kurz et al., 2017; Tatti et al., 2022). Thus, it is plausible to extrapolate that more demanding or interactive proprioceptive tasks might elicit stronger gamma responses, whereas simpler, passive movements may not generate significant changes in gamma oscillatory activity. This hypothesis aligns with the understanding that gamma oscillations support processes involving sensory integration and attention, which are more actively engaged in complex tasks (Gaetz et al., 2013; Spooner et al., 2020). The increment in gamma activation observed exclusively in children with CP, along with their poor proprioceptive performance, may indicate that these children might rely on compensatory neural mechanisms involving increased cognitive load to process proprioceptive input effectively (Busboom et al., 2024; Kurz et al., 2017).

Our study has several limitations that should be acknowledged for the adequate interpretation of these results. First, the sample size of children with CP and ASD was relatively small, which may limit the generalizability of our findings to the broader population of children with these conditions. Future studies with larger, more diverse samples are needed to confirm and extend these results. Second, while we controlled for various potential confounding variables, such as age and general cognitive abilities, individual differences in sensory processing and cognitive load responses may have influenced the outcomes. Future research should include more detailed assessments of these factors to better understand their role in proprioceptive and gamma oscillatory activity. Finally, the ecological validity of the findings is limited by the controlled laboratory environment. Although affective touch was shown to influence gamma activity and proprioceptive hits, real-world interactions involving dynamic, multimodal sensory inputs might yield different results. Future studies should incorporate more naturalistic settings and multimodal sensory stimuli to enhance the applicability of the findings. Despite these limitations, this study provides meaningful insights into the potential of affective touch to influence proprioceptive processing, particularly in individuals with CP. Further research is essential to explore the mechanisms underlying these effects and to evaluate how they can be harnessed in therapeutic settings.

In conclusion, his study contributes to the growing body of evidence linking emotional and sensory experiences with motor and proprioceptive processing in children with different neurodevelopmental conditions. Our findings underscore the significant impact of affective touch on proprioceptive processing and performance, emphasizing the critical role of affective touch in sensory integration for those populations with altered sensory pathways and suggesting potential pathways for therapeutic interventions aimed at enhancing sensory and motor performance in children with proprioceptive impairment.

## Data Availability

The raw data supporting the conclusions of this article will be made available by the authors, without undue reservation.
